# Spatio-temporal distribution of COVID-19 cases and tuberculosis in four provinces of Sumatra Islands, Indonesia

**DOI:** 10.1186/s12889-025-21754-z

**Published:** 2025-02-10

**Authors:** Arif Saputra, Wit Wichaidit, Zurnila Marli Kesuma, Virasakdi Chongsuvivatwong

**Affiliations:** 1 Department of Epidemiology, Faculty of Medicine, Prince of Songkla University, Hat Yai, 90110 Thailand; 2 Department of Statistics, Faculty of Mathematics and Natural Sciences, Universitas Syiah Kuala, Banda Aceh, 24415 Indonesia

**Keywords:** Spatio-temporal, COVID-19, Tuberculosis, Lag analysis, Indonesia

## Abstract

**Background:**

The COVID-19 pandemic disrupted national tuberculosis programs in high-burden countries. We hypothesize that COVID-19 occurrence had a delayed effect on tuberculosis case reports from the Indonesia Ministry of Health, also known as the tuberculosis case notification. The objectives of this study are: (1) to describe the potential effect of the reported COVID-19 cases on the spatial distribution of tuberculosis in four provinces of Sumatra Islands (Aceh, North Sumatra, West Sumatra, and Riau Provinces), Indonesia; (2) to estimate the temporal lag effect of the occurrence of COVID-19 on tuberculosis case notifications.

**Methods:**

We retrieved data from the Indonesian Ministry of Health and the Indonesia COVID-19 Task Force. We also examined the monthly tuberculosis and COVID-19 case notifications. We identified time series clusters of tuberculosis case notifications and used lag non-linear model to assess the delayed effect of the occurrence of COVID-19 cases on tuberculosis case notifications.

**Results:**

The secondary data included 217,593 tuberculosis case notifications (January 2019 to December 2022) and 373,671 reported COVID-19 cases (January 2020 to December 2022). Time series cluster analysis revealed 5 clusters each for monthly tuberculosis case notifications and monthly reported COVID-19 cases. There was a negative association with a 0-month lag in more than 10,000 reported COVID-19 cases (RR = 0.95, 95%CI: 0.91–0.98).

**Conclusions:**

The findings suggested that Indonesia’s national tuberculosis program in four provinces of Sumatra Island was disrupted during the COVID-19 pandemic. Lag analysis showed that COVID-19 case occurrence had an immediate effect on tuberculosis case notifications.

**Clinical trial:**

Not applicable.

**Supplementary Information:**

The online version contains supplementary material available at 10.1186/s12889-025-21754-z.

## Background

The World Health Organization (WHO) declared the coronavirus disease (COVID-19), caused by severe acute respiratory syndrome coronavirus 2 (SARS-Cov-2), a global pandemic in March 2020 [1–3]. The pandemic disrupted public health programs worldwide, including tuberculosis programs, which remain a major global health concern [4, 5]. In Indonesia, a high-burden country, the pandemic noticeably affected tuberculosis case detection [6]. Four provinces in the upper part of the Sumatra Islands of Indonesia, namely Aceh, North Sumatra, West Sumatra, and Riau, are part of Indonesia where the tuberculosis burden has been the highest [7]. They were also heavily affected by COVID-19 pandemic compared to the remaining parts of the country [8].

Disruption to tuberculosis case detection was partly attributed to the implementation of COVID-19 prevention measures, such as lockdowns, restrictions on movement, and the reallocation of healthcare resources toward pandemic response efforts. These measures may have reduced access to diagnostic and treatment services for tuberculosis patients [9–12]. Furthermore, decision-makers in public health may require time for data processing and review of empirical evidence. Consequently, the response to changes in tuberculosis case trends during the pandemic may have been delayed, compounding the challenges of addressing tuberculosis in the context of a concurrent COVID-19 crisis. Furthermore, previous studies showed that the COVID-19 pandemic attributed to a delayed effect of tuberculosis case detection can lead to an increase in community transmission [13]. Previous studies showed that an external disturbance might influence the healthcare-seeking behavior of tuberculosis-suspected individuals by implementing the Distributed Lag Non-linear Model (DLNM) [14–17]. However, those studies only covered environmental factors as the external disturbance. It is essential to understand whether COVID-19 is attributed to a delayed effect on tuberculosis case detection for the exact purpose of confronting a rebound effect of tuberculosis after the pandemic is over.

We hypothesize that the occurrence of COVID-19 had a delayed effect on tuberculosis case notification, as disruptions to tuberculosis detection and treatment likely persisted even as the immediate burden of COVID-19 fluctuated. Addressing this knowledge gap could contribute to future contingency planning by national tuberculosis programs and improve their resilience [18]. Thus, the objectives of this study are: (1) to describe the potential effect of reported COVID-19 cases on the spatial distribution of tuberculosis in four provinces of Sumatra Islands (Aceh, North Sumatra, West Sumatra, and Riau Provinces), Indonesia, and; (2) to estimate the delayed effect of the occurrence of COVID-19 on tuberculosis case notifications.

## Methods

### Study design and setting

We conducted an ecological study in 4 provinces of Sumatra Islands of Indonesia: Aceh, North Sumatra, West Sumatra, and Riau. These four provinces altogether consist of 87 districts covering more than 33 million population in 2024 [19].

### Data source, data extraction, data management, and data cleaning

We received the monthly tuberculosis case notification and the monthly COVID-19 case reports from the *Sistem Informasi Tuberkulosis* (SITB) Indonesia Ministry of Health [20] and the official website of Indonesia’s National COVID-19 Task Force [8], respectively. We extracted the monthly tuberculosis case notification data for each district from January 2019 until December 2022 to capture the tuberculosis condition before and during the pandemic, and the monthly COVID-19 reported cases data for each district from January 2020 until December 2022. COVID-19 was first reported in Indonesia in March 2020, thus we did not have data for COVID-19 in 2019 [21]. We combined both data sets into one based on the month and year of reporting, and translated the header row and the cells into English. We treated cells with “N/A”, “unknown”, and “not recorded” or empty cells as those with missing values. To ensure the DLNM model captured the delayed effect effectively, we removed the tuberculosis case notifications in 2019.

### Data analysis

We transformed the monthly tuberculosis case notifications and monthly reported COVID-19 cases into logarithmic scales to accommodate the large gap in the number of cases between the two diseases. We then created an electronic map of the study area and geocoded the area by district by estimating the center point from the district’s shape. We then used time series cluster data analysis to group time series data of various regions within the study area and detect groups or clusters of districts.

As the arrival of COVID-19 incidence surge might have had a delayed effect before the tuberculosis case notification was changed, the correlations between these two monthly values were examined with different lag (delayed effect) values. The value which gave the highest absolute value of Spearman’s correlation coefficient was the answer to the delayed time (DLNM model) [22]. This lag value was used for further analysis of predicting the decline of tuberculosis case notifications from the surge of COVID-19 incidence [22]. The association between the reported COVID-19 cases and (optimal lag value) of subsequent tuberculosis case notifications was shown by relative risk (RR) with a 95% confidence interval (CI).

### Ethical consideration

This study received ethical approvals from the Human Research Ethics Unit of the Faculty of Medicine, Prince of Songkla University, Hat Yai, Thailand (REC.66-245-18-2) and from the Research Ethics Committee of the Faculty of Nursing, Universitas Syiah Kuala, Banda Aceh, Indonesia (Research code: 113009120923).

## Results

### Distribution of the case reports

We obtained a total of 217,593 tuberculosis case notifications from January 2019 to December 2020, and 373,671 COVID-19 case records from January 2020 to December 2022, covering the 4 study provinces for both cases. The lowest number of tuberculosis cases was recorded in 2020, whereas the highest number was observed in 2022 (Table 1). The lowest number of COVID-19 cases reported was in 2022, while the highest was in 2021. Notably, West Sumatra reported only 93 cases of COVID-19 in 2022, while other provinces reported more than 6,000 cases in the same year (Table 1).

**Table 1:** Distribution of tuberculosis and COVID-19 cases by year and province

Province	Tuberculosis cases (%)				COVID-19 cases (%)			
	2019	2020	2021	2022	2019	2020	2021	2022
Aceh	8404 (14.5)	6752 (16.1)	7095 (15.1)	10,820 (15.3)	-	8123 (11.2)	28,383 (12.3)	6412 (9.2)
North Sumatra	27,897 (48.2)	20,735 (49.4)	22,514 (47.9)	34,576 (48.9)	-	16,589 (22.8)	70,894 (30.7)	41,070 (58.8)
West Sumatra	10,910 (18.9)	5755 (13.7)	8118 (17.3)	12,741 (18.0)	-	23,441 (32.2)	33,212 (14.4)	93 (0.1)
Riau	10,634 (18.4)	8758 (20.9)	9322 (19.8)	12,562 (17.8)	-	24,660 (33.8)	98,527 (42.7)	22,267 (31.9)
**Total cases**	**57,845**	**42,000**	**47,049**	**70,699**	**-**	**72,813**	**231,016**	**69,842**

COVID-19: coronavirus disease 2019

**Fig. 1:**
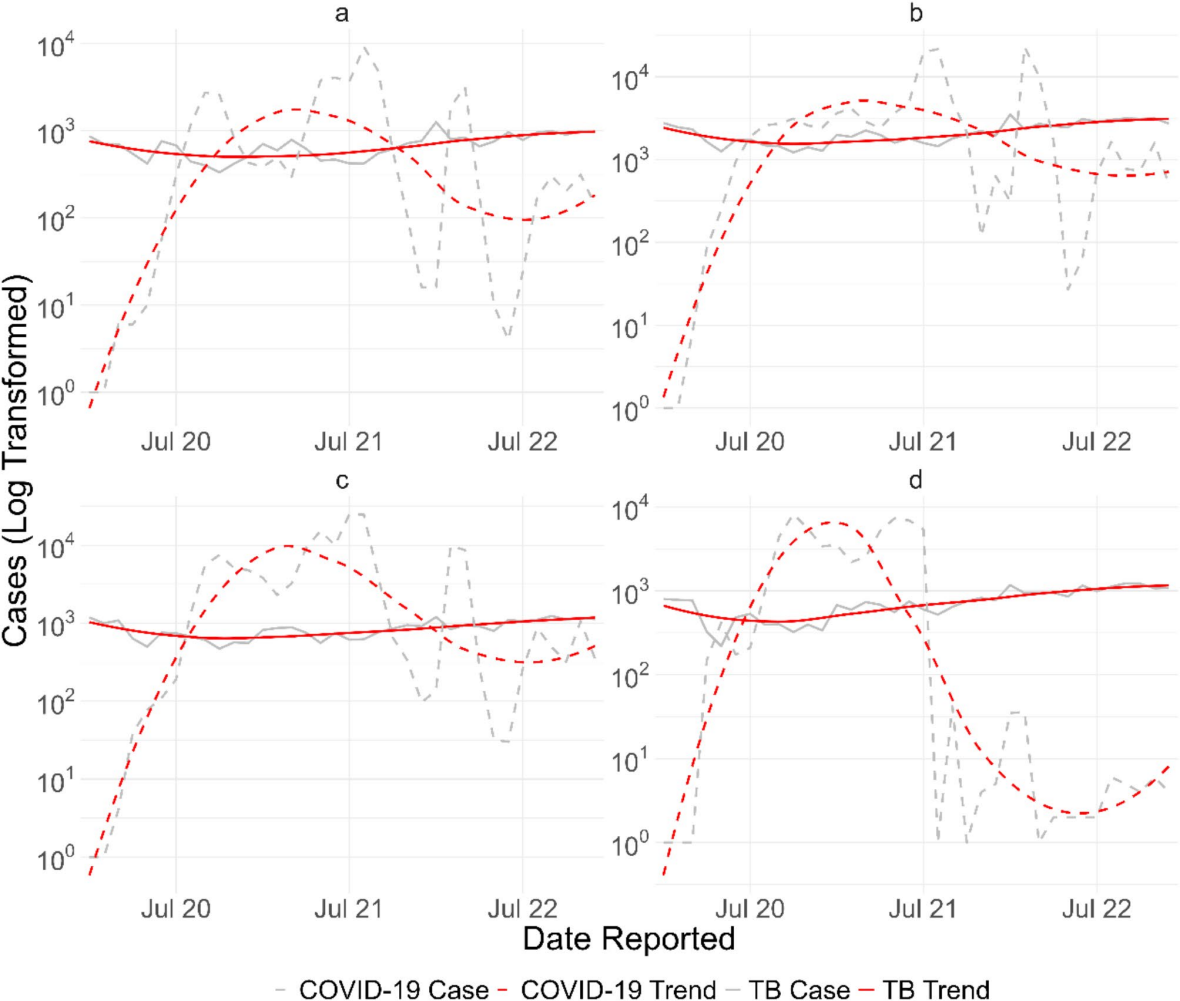
Distribution of monthly tuberculosis case notifications (solid line) and COVID-19 cases (dotted line) in (**a**) Aceh; (**b**) North Sumatra; (**c**) West Sumatra; (**d**) Riau

Trends data on both monthly tuberculosis and COVID-19 case notifications (Fig. 1) showed that as COVID-19 cases fluctuated, tuberculosis case notifications remained relatively stable. However, the actual number of tuberculosis case notifications on the log scale suggested that variations indeed existed for tuberculosis case notifications, but were dwarfed by variations in the number of COVID-19 cases. When we displayed tuberculosis case notifications alone (Supp. Figure 1), we observed a similar pattern of changes in all provinces: there were declining trends in notifications from 2019 to late 2020, followed by rising trends from early 2021 to late 2022. This implies that although there were negative correlations between the number of COVID-19 cases and tuberculosis case notifications, the trends in the tuberculosis notifications might have started before the COVID-19 pandemic began in the area. The effect of COVID-19 on tuberculosis case notifications was inconclusive according to the Figures.

**Fig. 2:**
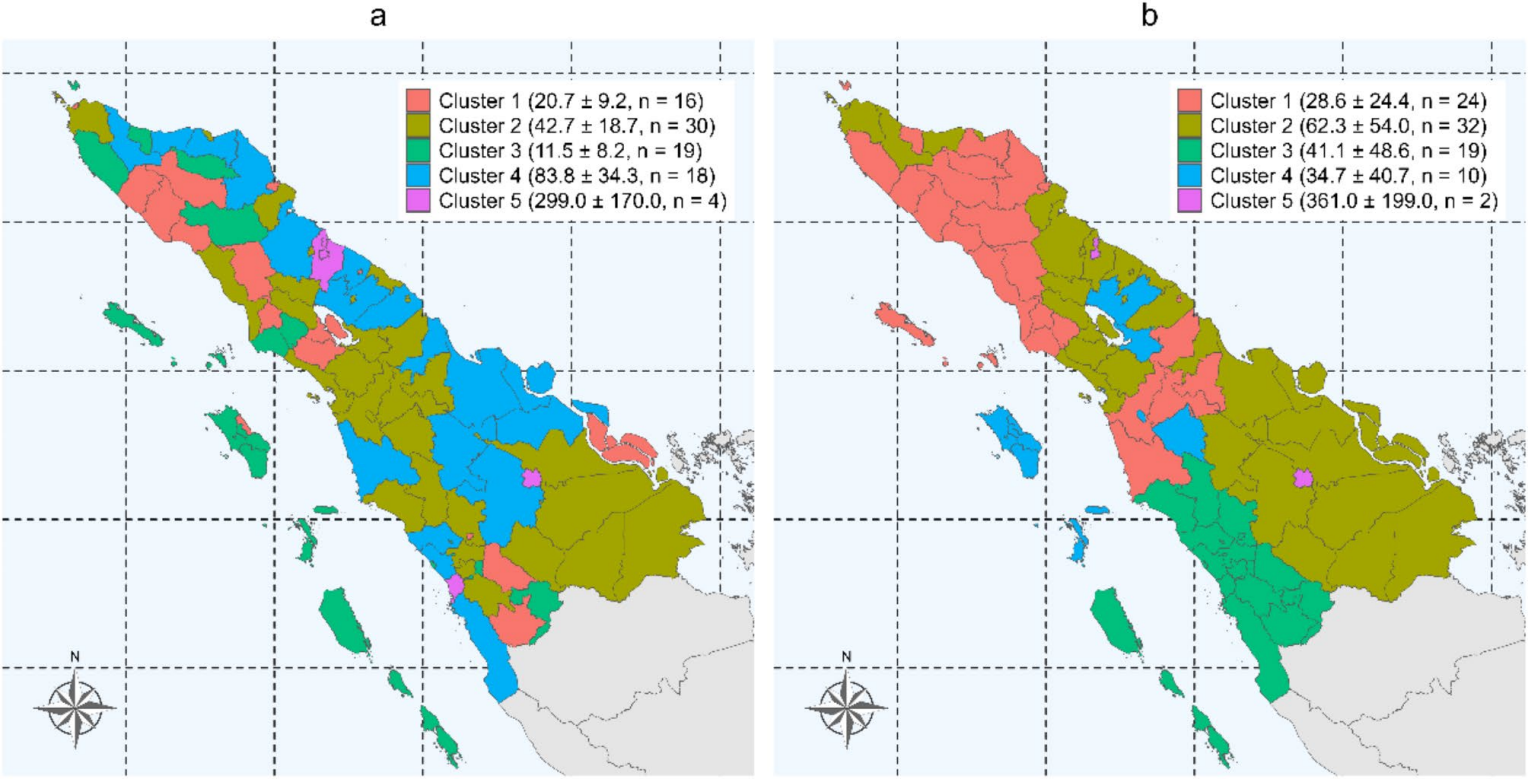
Distribution of the time series cluster analysis for (**a**) monthly tuberculosis case notifications and (**b**) monthly COVID-19 reported cases

We identified 5 clusters of monthly case records for both diseases in four provinces of the Sumatra Islands, Indonesia by using the time series cluster analysis (Fig. 2). Regarding tuberculosis, we identified districts in Cluster 3 as having the lowest distribution of monthly tuberculosis case notifications, and districts in Cluster 5 as having the highest distribution (Fig. 2.a). Districts on the northern coast of Aceh Province exhibited a shared temporal pattern of tuberculosis case notifications in Cluster 4. Islands districts within the Sumatra Islands demonstrated a similar pattern in Cluster 3. Big cities also shared similar temporal patterns of tuberculosis case notifications in Cluster 5. Regarding COVID-19, we identified districts in Cluster 1 and Cluster 5 as those with the lowest and highest distribution of reported monthly COVID-19 cases, respectively (Fig. 2.b). Most districts within the same province shared a similar monthly distribution of COVID-19 cases. The temporal patterns of each district within the cluster for each disease are shown in Supp. Figure 2. Overall, tuberculosis case notifications seemed to be more scattered and less volatile or fluctuated compared to reported COVID-19 cases.

### Lag analysis

**Fig. 3:**
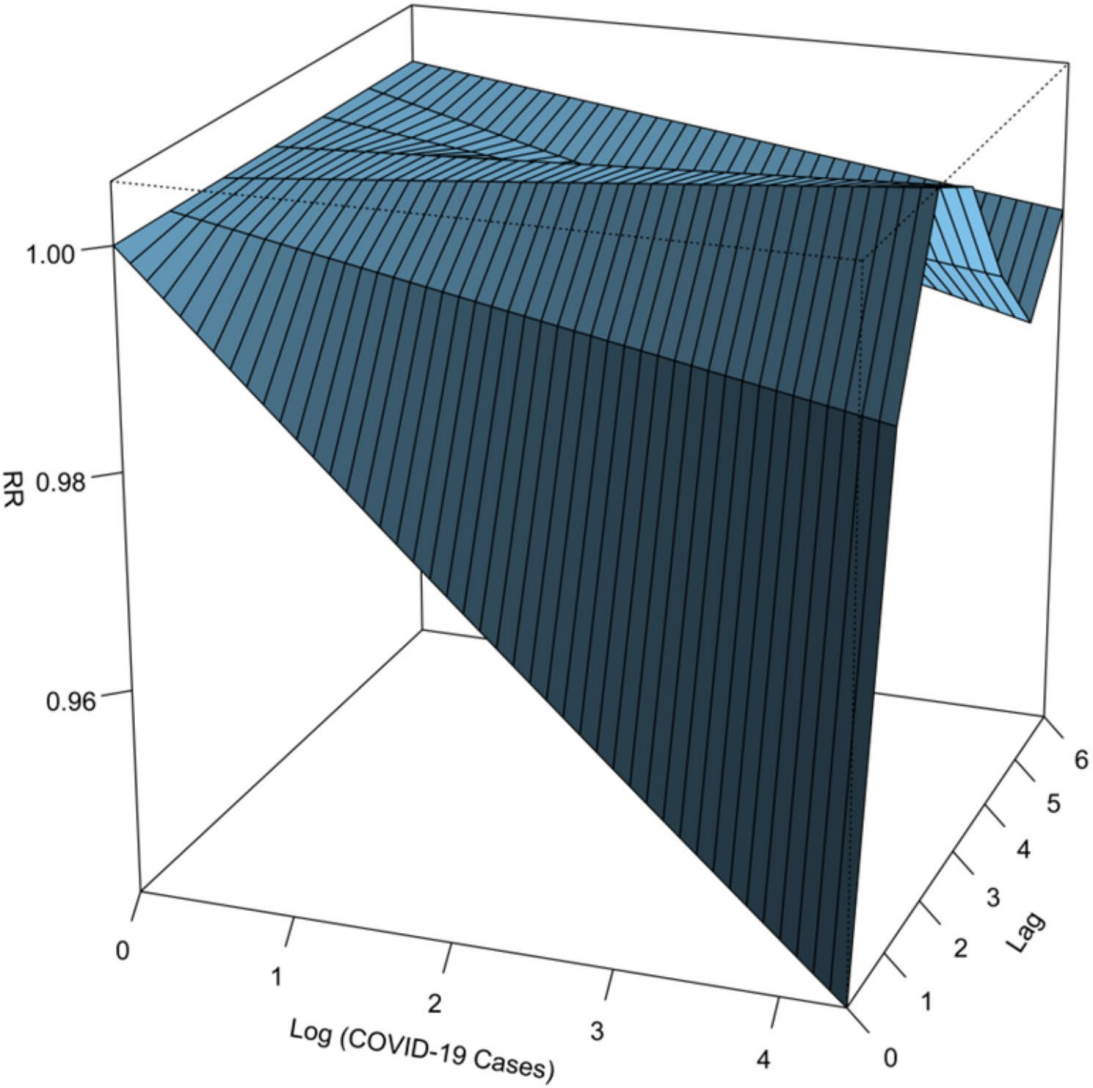
Three-dimensional relationship between log-transformed monthly COVID-19 cases, log-transformed monthly tuberculosis case notifications, and lag of 6 months

Spearman correlation indicated a non-significant negative relationship between monthly notified tuberculosis cases and COVID-19 cases (R_spearman_ = -0.14; p-value > 0.05). The distributed lag non-linear model (DLNM) with a three-dimensional plot (Fig. 3) showed that the association was negative at shorter lags but became positive at longer lags. There were significant negative associations at a 0-month lag, with the strongest negative association occurring when there were more than 10,000 COVID-19 cases reported (RR = 0.91, 95%CI: 0.89–0.92).

**Fig. 4:**
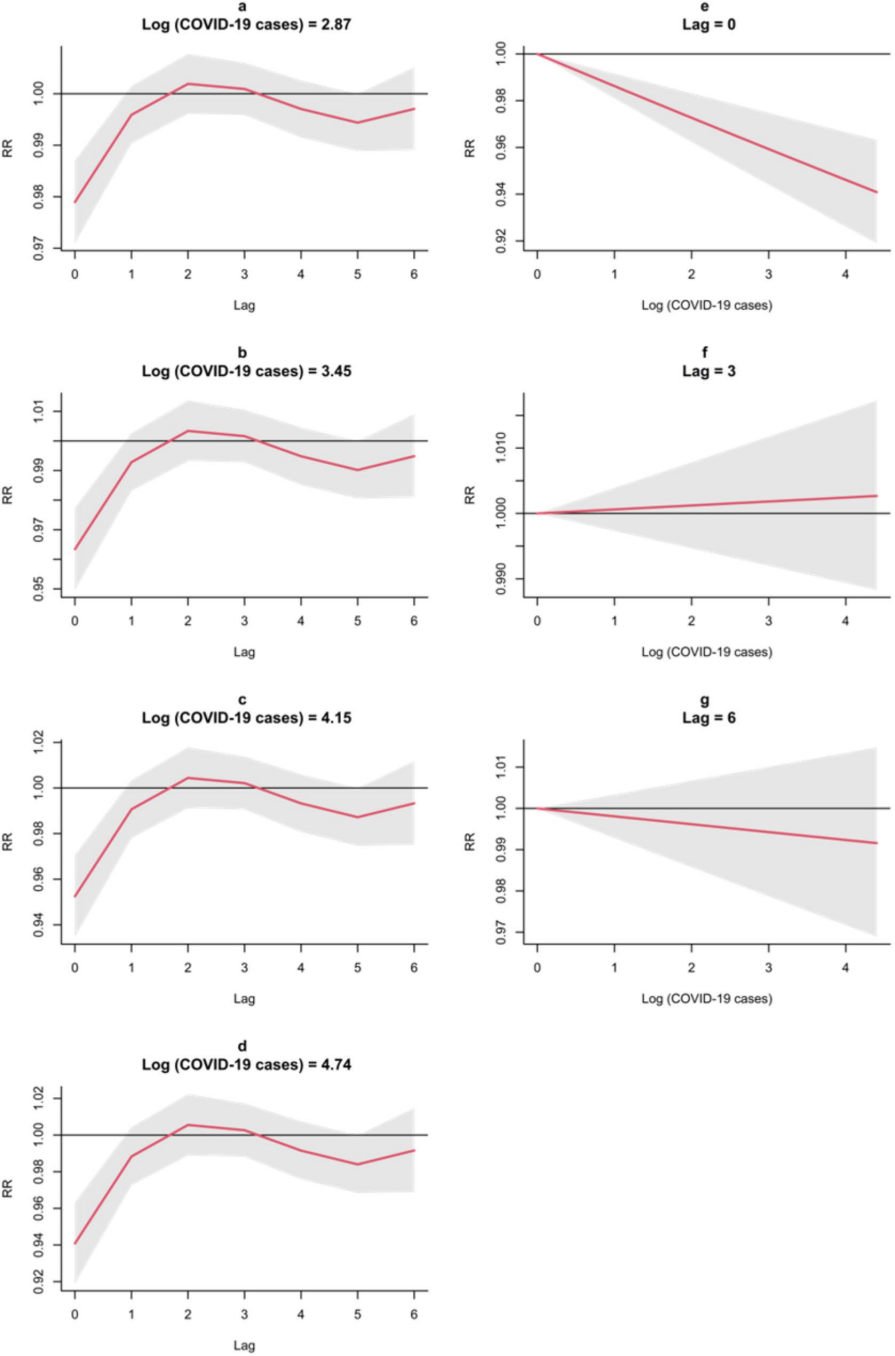
The plots of RR by lag at 25th, 50th, and 75th quartile of log-transformed monthly reported COVID-19 cases (**a**-**d**) and RR by log-transformed monthly reported COVID-19 cases (**e**-**g**)

Plots of specific predictors of monthly reported log-transformed COVID-19 cases (corresponded to the 25th, 50th, 75th quartiles and maximum number of COVID-19 cases) and specific lags (0, 3, and 6 month-lags) (Fig. 4) showed a consistent pattern increased risk of tuberculosis throughout the lag period as measured by relative risk, with the lowest association observed in the 0-month lag. The strongest negative association was found during the 0-month lag indicating that the effect of the COVID-19 surge on the decline of tuberculosis case notifications was immediate or without any delay. Furthermore, at specific lags, the association gradually declined during the 0-month lag and 3-month lag, while it gradually increased during the 6-month lag.

The modified model with − 2-month and − 1-month as the minimum lag (Supp. Figure 3) showed a three-dimensional association with a minimum lag of -1-month and − 2-month lags. Adjusting the minimum lag to negative revealed delayed effects occurring 1 month after COVID-19 cases were reported. In the − 1-month model, the lowest estimated RR was 0.97 (95%CI: 0.95–0.99) in the 1-month lag, coinciding with the maximum number of monthly reported COVID-19 cases. While in the − 2-month model, the lowest estimated RR was 0.98 (95%CI: 0.96–0.99) under similar conditions. However, compared to the initial model where immediate effects of COVID-19 cases were detected, the changes in the association during 0-month and 1-month lags in the modified models were not as significant.

## Discussion

In this secondary data analysis, we described the distribution of tuberculosis and COVID-19 case notifications in four provinces of Sumatra Island and estimated the temporal lag effect of COVID-19 on tuberculosis case notifications in the mentioned region. We were able to find hotspot by space and time. We also found a reduction in tuberculosis case notifications in every province of the study area, which suggested a disruption in tuberculosis case findings, particularly from March to December 2020. The findings suggested that the health systems should be developed to be more resilient to the pandemic. Otherwise, the systems will be immediately disrupted and people will be immediately neglect the systems and tuberculosis will be rapidly expanded.

We found a sharp rise in the number of detected COVID-19 cases in the fourth quartile of 2020, which coincided with a stall in tuberculosis case detection. The rise in reported COVID-19 cases could be attributed partly to the efforts of public health officials in conducting active surveillance (contact tracing, testing, and treatment for COVID-19) [23, 24]. The drop in tuberculosis case detection could be partially attributed to focus-shifting among healthcare workers. However, COVID-19 cases data in our study were based only on reported cases (i.e., from passive surveillance) and not from active surveillance data. The possibility of selection bias should be considered as a caveat in the interpretation of these study findings.

We found districts bordering each other shared similar temporal patterns of monthly tuberculosis case notifications, with the exceptional of big cities with better socioeconomic conditions, such as Medan and Deli Serdang in North Sumatra, Pekanbaru in Riau and Padang in West Sumatra. Those big cities with large populations are prone to tuberculosis transmission yet have better access to healthcare facilities [25, 26]. Surprisingly, we found that the temporal pattern of a populous city in Aceh, namely Banda Aceh, did not share a similar pattern with those cities. This might be due to the notable gap in the monthly tuberculosis case notification in Banda Aceh and those cities.

We conducted a novel analysis to examine the lag effect of COVID-19 on tuberculosis. We found that COVID-19 case reports had an immediate effect (0-month lag) on tuberculosis case notifications. The findings suggested that Indonesia’s tuberculosis program was susceptible to disruptions during the pandemic. Although previous studies found a delayed effect of meteorological variables on respiratory diseases [16, 17], confounding could not be precluded from our findings. The observed stall in tuberculosis case findings could have been attributed to unmeasured external factors, such as the implementation of disease control measures, as highlighted in a qualitative study conducted by some of the study’s investigators [6]. When we modified the minimum lag into − 1 and − 2 months, we observed delayed effects during the first month of the pandemic in both models, but the associations at the 1-month lag were nearly identical to those at the 0-month lag. Therefore, the baseline model with a minimum lag of 0 was deemed more appropriate for estimating the association between COVID-19 and tuberculosis cases. Delays in healthcare-seeking behavior during the pandemic may be attributed to factors such as the fear of SARS-Cov-2 infection at work and the complex procedures in accessing health services [27]. Future studies should consider a detailed exploration of the nature of these disruptions to understand the nature of these apparently immediate-effect disruptions.

The strength of this study was in its novelty, being the first study to attempt to describe the potentially delayed effect of reported COVID-19 cases on tuberculosis case detection. The findings of this study could be useful in building resilient health systems that have the capacity to maintain essential healthcare services while addressing the challenges of new diseases and potential future pandemics. However, a number of limitations should be considered in the interpretation of the study findings. Firstly, the use of only reported case data and not data from active surveillance implies that potential selection bias could not be precluded from the study findings. Secondly, as the study data only included the distribution of reported or notified cases by province and period, unmeasured confounding could have accounted for parts of the observed findings. Thirdly, the results may be confounded by existing reporting systems, which were incomplete and bias toward underestimation of the real COVID-19 incidence. Furthermore, we only collected data from provinces on Sumatra Island, which had a significantly lower population density than neighboring Java Island. The lack of generalizability also presented an additional caveat in the interpretation of the findings.

## Conclusions

We analyzed secondary data on COVID-19 and tuberculosis case findings and found that Indonesia’s national tuberculosis program in four provinces of Sumatra Island was prone to disruption by the COVID-19 pandemic, with a noticeable lag effect. However, the use of reported cases instead of active surveillance data and issues regarding generalizability should be considered as caveats in the interpretation of the study findings.

## Electronic supplementary material

Below is the link to the electronic supplementary material.

Supplementary Material 1

## Data Availability

Datasets used in this analysis are available from the corresponding author upon reasonable request.

## Abbreviations

CI: Confidence interval
COVID-19: Coronavirus disease 2019
DLNM: Distributed lag non-linear model
N/A: Not available
RR: Relative risk
RT-PCR: Reverse transcription polymerase chain reaction
SARS-Cov-2: Severe acute respiratory syndrome coronavirus 2
