# Supplementary material for: Spatio-temporal distribution of COVID-19 cases and tuberculosis in four provinces of Sumatra Islands, Indonesia
Source: BMC Public Health. 2025 Feb 10;25:529. doi: 10.1186/s12889-025-21754-z (PMC11809051; doi:10.1186/s12889-025-21754-z)
Supplement: Supplementary file 1 — Supplementary Material 1 [file 12889_2025_21754_MOESM1_ESM.docx]

# Supplementary File

# Spatio-temporal distribution of COVID-19 cases and tuberculosis in four provinces of Sumatra Islands, Indonesia

Arif Saputra ^1^ *; Wit Wichaidit ^1^ *; Zurnila Marli Kesuma ^2^ *; Virasakdi Chongsuvivatwong ^1^ *

* These authors contributed equally to this work

^1^ Department of Epidemiology, Faculty of Medicine, Prince of Songkla University, Hat Yai 90110, Thailand.

^2^ Department of Statistics, Faculty of Mathematics and Natural Sciences, Universitas Syiah Kuala, Banda Aceh 24415, Indonesia.

# Supplementary Figures


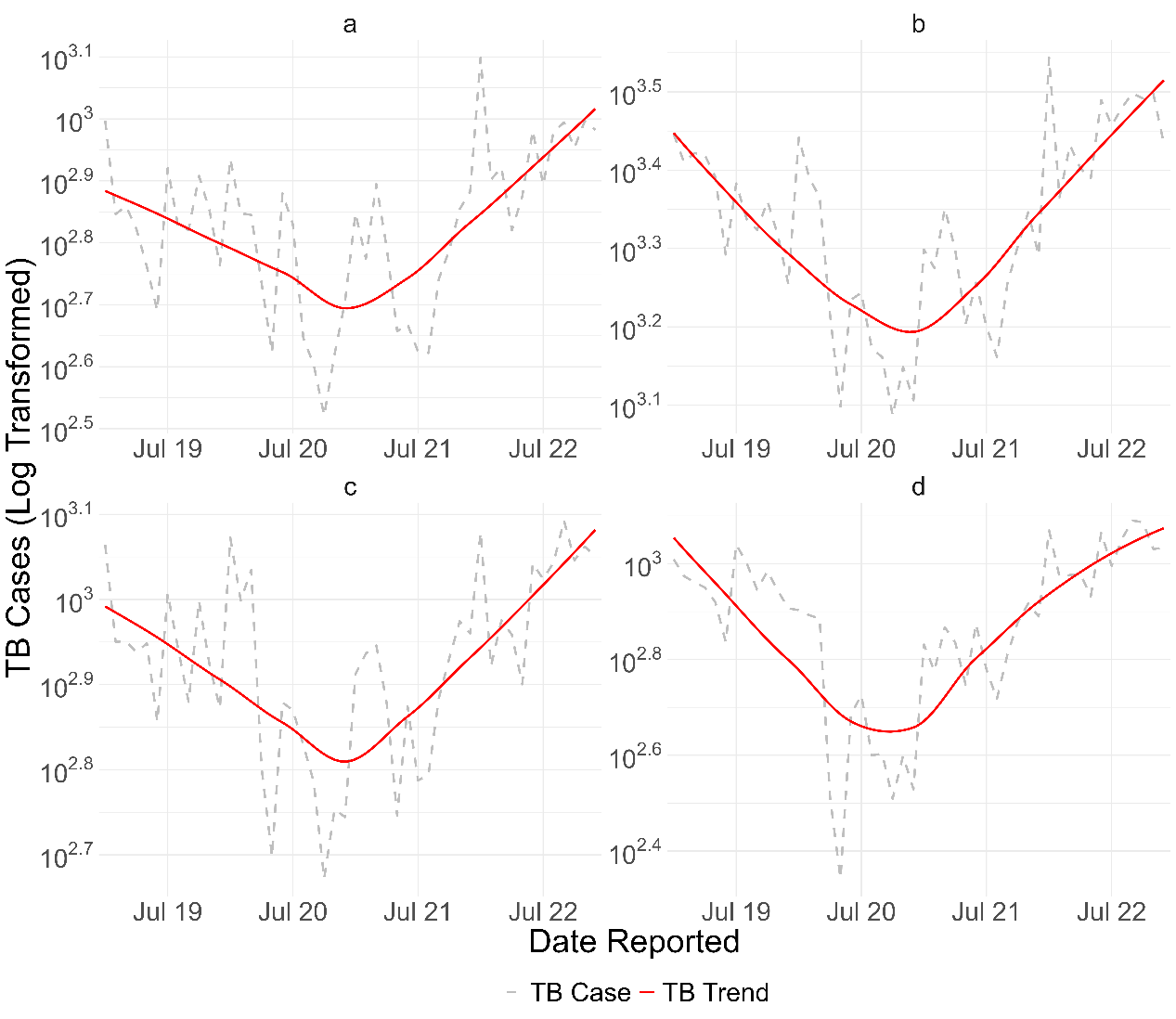


Supp. Figure 1. Distribution of monthly tuberculosis case notification (grey line) and smoothing trend (red line) in logarithm scale in (a) Aceh; (b) North Sumatra; (c) West Sumatra; (d) Riau


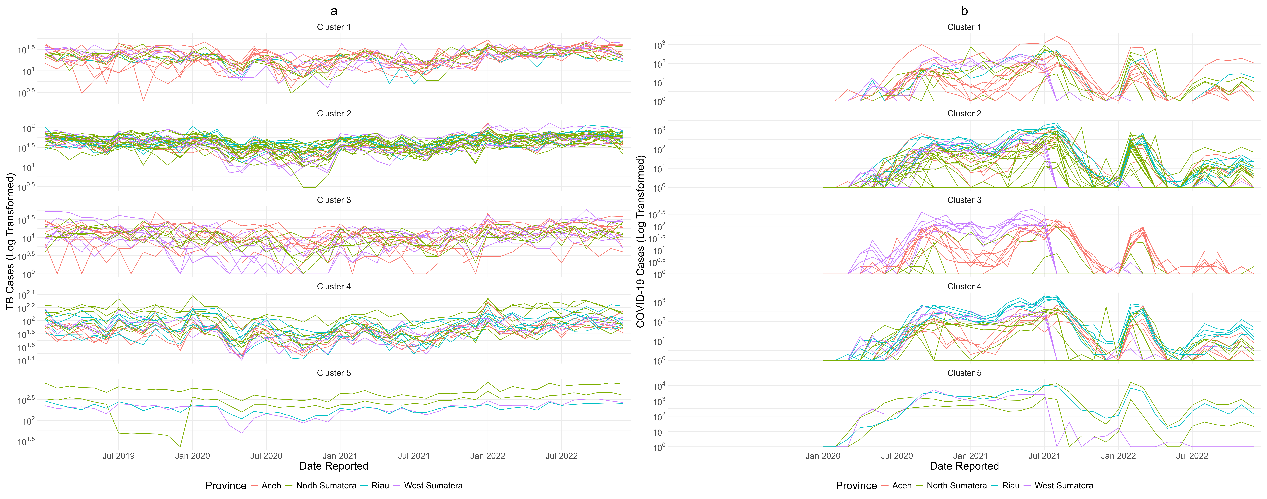


Supp. Figure 2. Temporal pattern of monthly distribution of reported cases by time series clusters for: (a) tuberculosis and (b) COVID-19





Supp. Figure 3. Three-dimensional relationship between log-transformed monthly COVID-19 cases and log-transformed monthly tuberculosis case notifications in the (a) minimum lag of -1 months and (b) minimum lag of -2 months
